# P-576. Real-world Experience with the Two-Drug Regimen Dolutegravir/Lamivudine for the Treatment of HIV-1 among Vulnerable People Living with HIV in Canada: Preliminary Results from a Chart Review Study

**DOI:** 10.1093/ofid/ofae631.774

**Published:** 2025-01-29

**Authors:** Emmanuelle Huchet, Joann K Ban, Adenike R Adelakun, Katherine Osenenko, Isabelle Hardy, Elaine Stewart, Michael McKimm, Jean-Francois Fortin, Madeleine Crabtree, Karissa Johnston, Brian Conway, V Paul DiMondi

**Affiliations:** Clinique Médicale L'agora, Montréal, Quebec, Canada; GlaxoSmithKline Canada Inc., Mississauga, Ontario, Canada; GSK, Montréal, Quebec, Canada; Broadstreet HEOR, Vancouver, British Columbia, Canada; ViiV Healthcare ULC, Montréal, Quebec, Canada; ViiV Healthcare ULC, Montréal, Quebec, Canada; ViiV Healthcare, North Vancouver, British Columbia, Canada; ViiV Healthcare ULC, Montréal, Quebec, Canada; Broadstreet HEOR, Vancouver, British Columbia, Canada; Broadstreet HEOR, Vancouver, British Columbia, Canada; Vancouver Infectious Diseases Centre, Vancouver, British Columbia, Canada; ViiV Healthcare, North Vancouver, British Columbia, Canada

## Abstract

**Background:**

Compared to the general population of people living with HIV, vulnerable people, such as those who use drugs, are disproportionately affected by HIV, and are predisposed to lower adherence to antiretroviral therapies (ARTs), which may result in poorer virologic suppression, worse health outcomes, and higher HIV transmission rates. Some may benefit from simpler single-tablet regimens that are effective, well tolerated, and limit exposure to unnecessary medications. We evaluated clinical outcomes in vulnerable people living with HIV who switched to the single-tablet, 2-drug regimen dolutegravir/lamivudine (DTG/3TC).

**Methods:**

This ongoing chart review study included people living with HIV (≥ 18 years) in Canada who switched to DTG/3TC between 09/09/2019 and 31/05/2023, and had ≥ 1 of the following vulnerability criteria: recent drug use, opioid agonist use, recent or well-documented history of unstable housing or receiving social assistance, Indigenous identity, or ≥ 65 years of age with diminished autonomy (‘vulnerable senior’). Descriptive summary statistics were generated for demographic and clinical characteristics at baseline (≤ 12 months pre-switch), and viral load and CD4+ cell counts at 6 (± 2) and 12 (± 2) months post-switch. Interim results are reported for the ongoing study.

**Results:**

Across 5 sites, 20 eligible people were included (mean age: 49.9 ± 13.0 years; male: 80%; drug use: 80%; vulnerable senior: 20%). Simplification of ART was the predominant reason for switch to DTG/3TC (n=11, 55%). At the time of analysis, 80% (n=16) had at least 6 months of follow-up, and one discontinued DTG/3TC due to intolerance. At 6 months, of those with test results, 8/8 were virally suppressed (< 50 c/mL), and median (IQR) CD4+ cell count was 815 (122.5) cells/mm3. At 12 months, 11/12 were virally suppressed (< 50 c/mL), and median CD4+ cell count was 920 (180) cells/mm3 (n=10).

**Conclusion:**

Preliminary results suggest promising effectiveness outcomes among vulnerable people living with HIV who switch to DTG/3TC. These results support the continued expansion of this study to include more people.

**Disclosures:**

**Emmanuelle Huchet, MD**, AbbVie: Honoraria|Gilead Sciences: Advisor/Consultant|Gilead Sciences: Honoraria|Indivior: Advisor/Consultant|ViiV Healthcare: Advisor/Consultant **Joann K. Ban, PharmD, MSc**, GSK: employee **Adenike R. Adelakun, BPharm, MSc**, GSK: employee **Katherine Osenenko, MCPM**, Broadstreet HEOR: employee|ViiV Healthcare: Funding for the conduct of this work **Isabelle Hardy, PhD**, ViiV Healthcare: employee **Elaine Stewart, HBSc**, ViiV Healthcare: employee **Michael McKimm, BSc**, ViiV Healthcare: employee **Jean-Francois Fortin, MD**, ViiV Healthcare: employee **Madeleine Crabtree, MPH**, Broadstreet HEOR: employee|ViiV Healthcare: Funding for the conduct of this work **Karissa Johnston, MSc, PhD**, Broadstreet HEOR: employee|ViiV Healthcare: Funding for the conduct of this work **Brian Conway, MD**, AbbVie: Advisor/Consultant|AbbVie: Grant/Research Support|AbbVie: Honoraria|AstraZeneca: Advisor/Consultant|AstraZeneca: Grant/Research Support|AstraZeneca: Honoraria|Gilead Sciences: Advisor/Consultant|Gilead Sciences: Grant/Research Support|Gilead Sciences: Honoraria|GSK: Advisor/Consultant|GSK: Grant/Research Support|GSK: Honoraria|Indivior Canada: Advisor/Consultant|Indivior Canada: Grant/Research Support|Indivior Canada: Honoraria|Merck: Advisor/Consultant|Merck: Grant/Research Support|Merck: Honoraria|Moderna: Advisor/Consultant|Moderna: Grant/Research Support|Moderna: Honoraria|Sanofi Pasteur: Advisor/Consultant|Sanofi Pasteur: Grant/Research Support|Sanofi Pasteur: Honoraria|Seqirus: Advisor/Consultant|Seqirus: Grant/Research Support|Seqirus: Honoraria|ViiV Healthcare: Advisor/Consultant|ViiV Healthcare: Grant/Research Support|ViiV Healthcare: Honoraria **V. Paul DiMondi, PharmD**, GSK: Stocks/Bonds (Public Company)|ViiV Healthcare: Employee

